# A Narrative Review of European Registries for Skin Cancer: Where Are We and Where Should We Be?

**DOI:** 10.3390/cancers18030524

**Published:** 2026-02-05

**Authors:** Alexander Katalinic, Karima Hammas, Lukasz Taraszkiewicz, Marieke Louwman, Joanna Julia Bartnicka, Giorgia Randi, Manola Bettio, Andreas Stang, Emanuele Crocetti

**Affiliations:** 1 Institute for Social Medicine and Epidemiology, University of Lübeck, 23562 Lübeck, Germany; 2 German Network of Cancer Registries (GNCR), 23562 Lübeck, Germany; 3 Haut-Rhin Cancer Registry, Groupe Hospitalier de la Région de Mulhouse et Sud-Alsace (GHRMSA), 68100 Mulhouse, France; karima.hammas@ghrmsa.fr; 4 Greater Poland Cancer Registry, Greater Poland Cancer Center, 61-866 Poznan, Poland; lukasz.taraszkiewicz@wco.pl; 5 Polish National Cancer Registry, Maria Sklodowska-Curie National Research Institute of Oncology, 02-781 Warsaw, Poland; 6 Department of Research and Development, Netherlands Comprehensive Cancer Organization (IKNL), 3511 Utrecht, The Netherlands; m.louwman@iknl.nl; 7 Joint Research Centre (JRC), European Commission, 21027 Ispra, Italy; joanna.bartnicka@ec.europa.eu (J.J.B.); manola.bettio@ec.europa.eu (M.B.); 8 Joint Research Centre (JRC), Piksel, Internal Service Provider at European Commission, 21027 Ispra, Italy; giorgia.randi@ext.ec.europa.eu; 9 Institute of Medical Informatics, Biometry and Epidemiology (IMIBE), University Hospital Essen, 45147 Essen, Germany; imibe.dir@uk-essen.de; 10 Cancer Registry of North Rhine-Westphalia, 44787 Bochum, Germany; 11 Romagna Unit, Emilia-Romagna Cancer Registry, IRCCS Istituto Romagnolo per lo Studio dei Tumori (IRST) Dino Amadori, 47014 Meldola, FC, Italy; emanuelecrocetti@yahoo.com

**Keywords:** skin cancer, cutaneous melanoma, non-melanoma skin cancer, cancer registration, incidence, ECIS, ENCR

## Abstract

**Background**: European population-based cancer registries (PBCRs) provide the foundation for monitoring skin cancer, yet registration practices and coverage vary, particularly for non-melanoma skin cancer (NMSC). **Methods**: We conducted a narrative review combining descriptive analyses of European Cancer Information System (ECIS) outputs with evidence from the European Network of Cancer Registries (ENCR) Working Group on NMSC and from national reports. A targeted PubMed search (2015–2025) assessed scientific usage of European registry data. **Results**: Nearly 200 PBCRs operate across about 40 European countries, with heterogeneous structures and timeliness. The ECIS estimated 101,500 incident cutaneous melanomas (CM) in the European Union in 2022. Long-term data from Nordic countries show a tenfold increase in CM incidence over the last six decades, with recent plateauing in younger cohorts. NMSC registration remains inconsistent: some countries record both cutaneous squamous cell carcinoma (cSCC) and basal cell carcinoma (BCC), others record cSCC only, and several omit NMSC entirely. Consequently, Europe-wide NMSC figures are not available from the ECIS. Global estimates exclude BCC and understate the true burden, which is likely between 1 and 1.6 million incident cases annually in Europe. The PubMed search identified 538 European registry-based publications on skin cancer (2015–2025). **Conclusions**: Melanoma registration in Europe is robust, but NMSC remains under-registered. Priorities include harmonized definitions and counting rules, better integration of outpatient and pathology data, streamlined EU-level reporting, digital/AI-enabled case ascertainment, and sentinel regions to generate reliable NMSC estimates.

## Simple Summary

Skin cancer is the most common cancer in fair-skinned populations. Melanoma is routinely captured by European cancer registries, but non-melanoma skin cancers (NMSCs)—especially basal cell carcinoma—are inconsistently recorded or even omitted. This narrative review summarizes where European registries currently stand and where they should go. We describe coverage and data quality, show current melanoma incidence levels and trends, and assess how registry data are used in research. Melanoma incidence has risen for decades but appears to plateau in younger cohorts, while the true NMSC burden is substantially underestimated because of incomplete registration. We outline practical steps to improve completeness (e.g., including outpatient and pathology data), timeliness, and harmonization, and propose sentinel regions for robust NMSC estimates. Better, faster, and more comparable registry data are essential to guide prevention, service planning, and policy in Europe.

## 1. Introduction

Cutaneous melanoma (CM) and non-melanoma skin cancer (NMSC) together represent a major and growing public health burden in fair-skinned populations worldwide. According to estimates from the International Agency for Research on Cancer (IARC), approximately 330,000 individuals are newly diagnosed with CM each year, and around 59,000 die from the disease [1]. CM arises predominantly among populations of European ancestry, reflecting the interplay of genetic susceptibility, phenotypic traits such as fair skin, and cumulative exposure to ultraviolet (UV) radiation [2,3,4].

Over the past few decades, CM incidence rates have increased markedly in most Western countries, largely driven by lifestyle-related UV exposure, increased awareness, and improved diagnostic intensity [5,6,7]. However, in some regions—most notably Australia, New Zealand, and parts of Northern Europe—a plateau or even a slight decline in incidence has been observed [7,8,9], which might reflect the long-term success of prevention campaigns and changes in sun-protective behavior. Despite these favorable trends, the absolute number of new CM cases is expected to remain high due to demographic ageing and population growth. Global Cancer Observatory projections suggest a further rise in melanoma cases until 2040, particularly in middle- and older-aged groups [10].

While CM receives substantial attention, interestingly, the group of NMSC is only rarely considered, despite accounting for the largest number of new cancer cases in fair-skinned populations [11,12]. NMSC is a heterogeneous group of tumors that usually arise from the keratinocytes of the skin, connective tissue, or migrated cells. The most common keratinocytic cancers are squamous cell carcinoma (cSCC) and basal cell carcinoma (BCC) [13]. Despite their frequency, reliable global epidemiological data for NMSC are sparse. The IARC currently provides a crude worldwide estimate of approximately 1.2 million incident NMSC cases for the year 2022 [1]. This number, however, underestimates the true burden, as BCC—the most frequent NMSC—is not included, and multiple lesions per individual are not captured. Based on registry and clinical data from selected countries, the true number of new NMSC cases worldwide may exceed 5 million annually [12].

Accurate and complete population-based cancer registration, including melanoma and NMSC, is essential for understanding the burden of skin cancer, identifying trends, revealing spatial differences, evaluating primary and secondary prevention and treatment strategies, and guiding health policy. However, registration of skin cancer across Europe remains heterogeneous. While melanoma is recorded routinely by population-based cancer registries (PBCRs), the inclusion of NMSC varies substantially—from comprehensive reporting in some countries to partial or no recording in others.

This narrative review provides an updated overview of skin cancer registration in Europe. Specifically, it addresses the following questions:

- What is the current status of skin cancer registration in European population-based cancer registries?
- What are the main epidemiological findings for melanoma and NMSC derived from these data?
- How are registry data used in skin cancer research across Europe?

By identifying gaps and best practices, this review seeks to outline the way forward toward more complete, harmonized, and research-oriented skin cancer registration in Europe.

## 2. Materials and Methods

This article presents a narrative review synthesizing the current status of skin cancer registration, epidemiology, and registry-based research in Europe. The approach combines descriptive analysis of publicly available data sources with a structured literature review and expert experience.

### 2.1. Cancer Entities and Classification

Skin cancer includes a variety of different cancer diagnoses. This review distinguishes between invasive cutaneous melanoma (CM; ICD-10 C43) and invasive non-melanoma skin cancer (NMSC; ICD-10 C44), comprising mainly keratinocyte carcinomas such as cSCC and BCC, defined by their histology (according to the International Classification of Diseases for Oncology (ICD-O), ENCR Recommendation on Non-Melanoma Skin Cancer [14]).

### 2.2. Data Sources

Quantitative and qualitative data were retrieved mainly from the following sources:

- European Cancer Information System (ECIS) (hosted by the European Commission’s Joint Research Centre), which compiles observed and estimated incidence and mortality data from European population-based cancer registries [15].
- Global Cancer Observatory (GCO) (hosted by the International Agency for Research on Cancer), for global estimates of cancer incidence and mortality [16].
- ENCR Working Group on Non-Melanoma Skin Cancer (ENCR NMSC WG) of the European Network of Cancer Registries (ENCR) [17] and European Commission’s Joint Research Center, Ispra, Italy. This group analyzed data from European Cancer Registries with the aim of establishing harmonized rules for recording and reporting NMSC and standardized methods to produce estimates for Europe.

Where appropriate, national registry reports or corresponding web pages (e.g., from the Nordic countries (NORDCAN [18]), Germany, and others) were used to complement European-level summaries.

### 2.3. Literature Search

To assess the use of European cancer registry data for research, a targeted search of PubMed/MEDLINE was conducted to identify studies involving EU Member States and the United Kingdom published between 2015 and 2025. The following terms, which were prioritized for sensitivity, were used to search mainly in the title and abstract of publications: ((“skin neoplasms”[Mesh] OR “skin cancer” OR “melanoma” OR “non-melanoma skin cancer”) AND (“cancer registr*”[tiab] OR “population-based registry”[tiab]) AND (“Europe”[Mesh] OR “European Union”[Mesh] OR European[tiab] OR EU[tiab] OR Austria[tiab] OR Belgium[tiab] OR Bulgaria[tiab] OR Croatia[tiab] OR Cyprus[tiab] OR Czech Republic[tiab] OR Denmark[tiab] OR Estonia[tiab] OR Finland[tiab] OR France[tiab] OR Germany[tiab] OR Greece[tiab] OR Hungary[tiab] OR Ireland[tiab] OR Italy[tiab] OR Latvia[tiab] OR Lithuania[tiab] OR Luxembourg[tiab] OR Malta[tiab] OR Netherlands[tiab] OR Poland[tiab] OR Portugal[tiab] OR Romania[tiab] OR Slovakia[tiab] OR Slovenia[tiab] OR Spain[tiab] OR Sweden[tiab] OR England[tiab] OR Scotland[tiab] OR “Northern Ireland” [tiab] OR (Wales[tiab] NOT Australia) OR “united kingdom”[tiab])) AND (“2015”[Date—MeSH]: “3000”[Date—MeSH]).

Further keywords such as “incidence”, “therapy”, etc., were applied to categorize the search results.

### 2.4. Data Synthesis

Findings were synthesized descriptively by topic:

- Cancer registration in Europe—coverage, completeness, and heterogeneity of melanoma and NMSC data.
- Epidemiological results—incidence levels and time trends for melanoma and NMSC across European countries.
- Registry-based research—publication patterns, main research questions, and use of registry linkages.

The results are summarized qualitatively and illustrated by figures and tables derived from the ECIS, GCO, and national cancer registry sources.

## 3. Results

### 3.1. Cancer Registration in Europe

**Figure 1:**
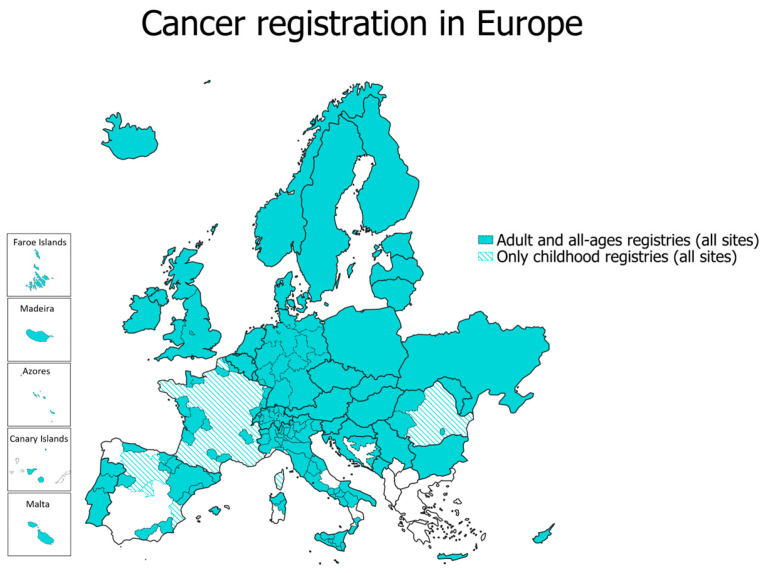
Coverage of cancer registration in Europe in 2025, based on the ENCR-associated registries list. Source: European Network of Cancer Registries [17]; https://www.encr.eu (accessed on 12 December 2025).

As of 2025, nearly 200 population-based cancer registries operate across approximately 40 European countries, covering large but not all parts of Europe (Figure 1). The organization and scope of cancer registration vary considerably between regions, reflecting differences in national health systems, legislation, and resource allocation.

In several south-eastern European countries, population-based cancer registration remains incomplete or absent; for instance, Greece still lacks full national coverage. In contrast, other nations operate comprehensive but decentralized systems composed of multiple regional registries, such as Italy (51 registries) and Germany (15 registries) [19]. Countries like Denmark, Norway, Slovenia, the Netherlands, Poland, and the United Kingdom operate centralized national registries that achieve nearly complete population coverage.

For the EU Member States, the ECIS estimated approximately 2.7 million new cancer cases in 2024, excluding NMSC. Age-standardized incidence rates varied substantially across countries. The highest overall cancer incidence rates were observed in Denmark, Croatia, the Netherlands, and Belgium, while the lowest rates were reported for Cyprus, Bulgaria, Luxembourg, and Austria (Table 1). These differences reflect not only variations in risk factor exposure but also differences in registration completeness and data quality among European cancer registries.

### 3.2. Coverage, Completeness, and Heterogeneity of Melanoma and NMSC Data

The registration of skin cancer across Europe shows substantial heterogeneity in coverage, coding practice, and data quality. Virtually all population-based cancer registries (all adult, ages, and sites, Figure 1) record cutaneous melanoma (ICD-10 C43), while the registration of non-melanoma skin cancer (ICD-10 C44) varies widely among and within countries.

In most European nations, melanoma registration is complete and standardized, supported by a high rate of anatomopathological examination, clear histopathological definitions, centralized pathology reporting, and mandatory notification systems. The Nordic countries, the United Kingdom, the Netherlands, and Slovenia have long-established melanoma data with country-wide, continuous, high-quality reporting. In contrast, as stated for cancer registration in general, few countries in south-eastern Europe still face incomplete coverage, limited access to electronic data, or delayed reporting to national databases. At the European level, epidemiological data on melanoma are available via the ECIS, where predicted incidence (“estimates”) for 2024 for the European Union (EU) and all member states alongside registered “real-world data” are presented. While estimates are updated every second year (the most recent ones refer to 2024), the update of real-world data is inherently slower, due to the time required to collect cancer registry data at the source, followed by subsequent reporting and EU-level validation. Currently, data up to 2020 are available in the ECIS for around 27 registries (21% of ECIS registries), and data up to 2022 or later are available for around 13 registries (10%).

The situation for non-melanoma skin cancer is considerably more complex. Within the ENCR NMSC working group, analyses were conducted on datasets from 86 CRs that submitted record-level data to the ECIS for the 2022 Data Call. Of these CRs, 52 (60%) submitted some SCC and BCC data at various levels of completeness, while a further 18 (21%) submitted only SCC data. In some European countries such as Belgium and Slovenia, cSCC, BCC, and other skin cancer cases are recorded. Other countries record only cSCC or only other skin cancers (e.g., Croatia). In Germany, Italy, and Spain, NMSC registration varies even within regions. Finally, countries such as Cyprus do not routinely include NMSC in population-based cancer registration. Consequently, there is currently no standardized data on NMSC available at the European level through the ECIS. The GCO released global estimates for NMSC, but these figures exclude BCC.

### 3.3. Epidemiological Results

According to estimates from the ECIS, approximately 104,000 new cases of cutaneous melanoma occurred in the EU in 2024. When excluding NMSC, melanoma accounts for roughly 3.9% of all malignant neoplasms diagnosed in the EU (Table 1).

**Figure 2:**
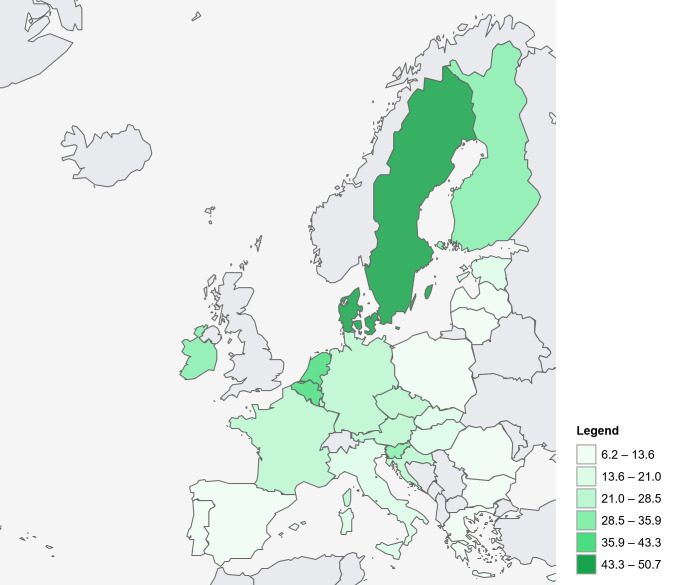
Estimated incidence of malignant melanoma of the skin in the year 2024 in the EU Member States: age-standardized rate (EU-2013) per 100,000 persons. Source: ECIS [15]; https://ecis.jrc.ec.europa.eu (accessed on 6 January 2026).

**Figure 3:**
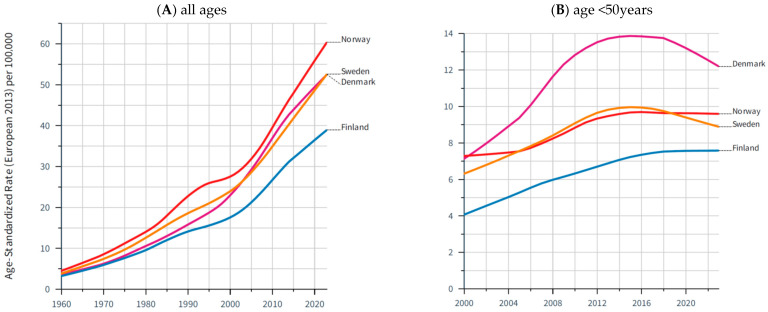
Incidence of melanoma of the skin in 4 Nordic countries: age-standardized rate (EU-2013) per 100,000 person-years. (**A**) Long-term trends for all ages; (**B**) mid-term trends for age <50 years. Lines are smoothed by the LOESS regression algorithm (bandwidth: 0.3/0.5). Source: NordCan (accessed on 22 October 2025) [18].

The highest melanoma incidence rates are estimated for the Nordic countries, the Netherlands, and Belgium, reflecting both high levels of UV exposure among fair-skinned populations and effective diagnostic surveillance. In contrast, southern European regions such as Portugal, Greece, and parts of Italy and Spain show lower age-standardized incidence rates (Figure 2). Long-term trend analyses from the Nordic Cancer Registries—representing some of the most complete and longest-running datasets in Europe—demonstrate a roughly tenfold increase in melanoma age-standardized incidence over the last six decades (Figure 3A) [18]. However, data from the last 20 years for younger age groups (age <50 years) indicate a plateau or even decline in melanoma incidence (Figure 3B). A recent analysis by the EUROCARE group on the survival of CM reveals a relative 5-year survival rate of 88% in Europe, with a range of about 90% in Central and Northern Europe, Ireland, and the UK, 86% in Southern Europe, and 75% in Eastern Europe [20].

For NMSC, no valid or comprehensive European estimates are available due to inconsistent registration practices. The GCO reported about 330,000 new NMSC cases in Europe in 2022 [1], but this figure excludes BCC and therefore captures only part of the true disease burden. As BCC typically occurs three to five times more frequently than cSCC [21,22], the real number of incident NMSC cases in Europe is likely in the magnitude of 1 to 1.6 million annually.

### 3.4. Skin Cancer Research Based on European Cancer Registries

A systematic PubMed search identified a steadily increasing number of publications using European population-based cancer registry data for skin cancer research. Between 2015 and 2025, a total of 538 publications were identified, originating predominantly from the Netherlands, the Nordic countries, the United Kingdom, Italy, Germany, and Ireland (Table 2). These regions are characterized by long-established cancer registries, nationwide or near-complete population coverage, and effective linkage with clinical and mortality databases.

The most frequently occurring keywords in the identified publications were incidence, prevalence, survival, and trends, reflecting the core epidemiological focus of registry-based research (Table 2). Studies addressing treatment modalities such as surgery, systemic anti-cancer treatment, or radiotherapy accounted for approximately 10% of all identified papers. Only about 100 publications explicitly mentioned NMSC, cSCC, or BCC, underscoring the limited use of registry data for these entities compared with melanoma.

## 4. Discussion

### 4.1. Summary of Key Findings

This review highlights major advances and persistent challenges in skin cancer registration across Europe. Nearly all European countries maintain population-based cancer registries, yet their structure, coverage, and timeliness remain heterogeneous. While the registration of cutaneous melanoma is largely complete and standardized, the registration of non-melanoma skin cancer remains inconsistent, with substantial gaps in many regions. Melanoma incidence continues to rise in most countries, albeit with indications of plateauing or decline among younger age groups. In contrast, the true burden of NMSC is unknown but likely falls within 1 to 1.6 million incident cases annually in Europe, underscoring a large hidden burden of disease. Finally, the use of cancer registry data for skin cancer research has increased markedly in the last decade, though it remains dominated by melanoma studies.

### 4.2. Strengths and Weaknesses of Current European Cancer Registration

Europe has made substantial progress toward comprehensive cancer registration. Since the establishment of the ENCR in 1990, cancer registries have benefited from the shared expertise through networking, training events, support in registry set-up, and ENCR recommendations that produce guidelines for harmonized registration. Comparable data on cancer burden and survival are provided by the ECIS or European projects, such as EUROCARE. The EU’s commitment to cancer control has led to funding for important initiatives, such as Europe’s Beating Cancer Plan (with one of the flagship projects, the European Cancer Inequalities Registry) and the recently launched CancerWatch Joint Action [23]. However, despite progress, there are still gaps in cancer registration coverage with “white spots” on the European map. Registration systems still differ markedly across countries, governance, resources, and legal frameworks.

A key limitation at the European level is timeliness. With a few exceptions, the data in the ECIS are not up to date. As of 2026, most ECIS datasets include only incidence data up to 2020, even though more recent data are available from many national registries. Here, the transfer of cancer registry data to the European level does not appear to be running optimally. Starting in 2026, however, the ECIS will implement a new approach to enable more agile, timely reporting through a streamlined process with annual deadlines for database updates. It remains to be seen whether this process will lead to more up-to-date data at the European level. The actual structural deficits limit comparability and slow progress in population-based cancer surveillance at the European level, not only for skin cancer. Continued investment in harmonizing registration, ensuring timely reporting, and automating data forwarding to the ECIS is essential to strengthening data quality and analytical capacity. Emerging technologies, including digital pathology, AI-based image recognition, and automated case ascertainment, may enhance the completeness and timeliness of registration. Pilot projects under the CancerWatch Joint Action [23] aim to demonstrate such integration, potentially setting new standards for future European cancer data infrastructure (European Health Data Space).

### 4.3. Interpretation of Skin Cancer Trends

The sustained increase in melanoma incidence across Europe mirrors cumulative UV exposure in fair-skinned populations, heightened diagnostic awareness, and the aging of post-war birth cohorts. The recent stabilization of rates among younger adults, particularly in Northern Europe, likely reflects the early impact of prevention and behavioral change campaigns such as Euromelanoma, SunSmart, and national UV awareness initiatives [2,8,24,25]. Whether these trends will be realized across Europe remains to be confirmed by more recent figures from cancer registries.

Despite these encouraging signs, the absolute burden of melanoma is expected to remain high due to demographic aging. Registry data provide a robust foundation for monitoring the success of prevention efforts, assessing survival trends, and evaluating early detection programs. However, detailed analyses require consistent recording of stage, histological subtype, and treatment variables, which remain incomplete in many registries, limiting the depth of outcome research [26,27]. In addition to methodological reasons arising from different cancer registration methods, epidemiological factors also play a causal role in the heterogeneity of skin cancer incidence across European countries, including differences in skin type distribution, sunbathing behavior, and cumulative UV dose.

Non-melanoma skin cancers represent the most common malignancy among fair-skinned populations, yet their incidence remains severely and systematically under-registered. There are only a few studies on the actual trends of NMSC in Europe, which show relatively stable incidences for cSCC and BCC in recent years [9,28,29,30]. Moreover, the shown numbers do not account for multiple primary lesions or recurrences, which are common among NMSC patients and further increase the clinical workload and economic impact of the disease [31,32,33]. The exclusion of BCC from many registries, combined with incomplete capture of cSCC, leads to severe underestimation of the true incidence and prevents a reliable assessment of morbidity, resource needs, and outcomes. The reasons are multifactorial: high case volumes, multiple primaries per patient, fragmented outpatient care, lack of mandatory reporting from pathology and dermatology practices to cancer registries, some treatments performed without previous histology confirmation, and, regarding BCC, a typically slow-growing tumor with very low metastatic potential and therefore low mortality. Given the clinical and economic relevance of NMSC, a better way to collect NMSC data is urgently needed [30]. It is doubtful whether comprehensive coverage of NMSC registration across Europe can be achieved. It seems more realistic to establish regional sentinel registries, as has been done in the USA within the framework of the Surveillance, Epidemiology, and End Results Program (SEER) for other cancer sites, that record NMSCs as completely as possible. Such sentinel data could be used to estimate the burden of NMSC in Europe.

### 4.4. Research Use of Cancer Registry Data

Cancer registry-based skin cancer research in Europe is expanding, reflecting growing interest in population-level data for epidemiology and health care research. Almost 50% of all publications worldwide from 2015 included European registries. Countries with well-integrated registries have produced most of the current evidence on incidence, survival, and temporal trends. In contrast, NMSC research remains sparse, constrained by incomplete data. Future progress depends on linking cancer registry data with electronic health records, pathology databases, and treatment registries.

### 4.5. The Limitations of This Review

This review followed a narrative rather than a systematic approach and therefore may not capture all relevant publications, but it included leading experts in the field of cancer registration. The quantitative summary of registry-based skin cancer publications was conducted without human screening of titles and abstracts and was limited to PubMed-indexed, English-language articles.

## 5. Conclusions

### 5.1. Where Are We?

While melanoma registration in Europe is well established and produces reliable, comparable data, non-melanoma skin cancer remains largely under-registered, resulting in a major blind spot in cancer surveillance. A coordinated European effort—combining technical harmonization, digital innovation, and sustained investment—is needed to achieve comprehensive, high-quality registration and to unlock the full potential of cancer registry data for research, prevention, and cancer control.

### 5.2. Future Direction—Where Should We Be?

To fully realize the potential of cancer registries for skin cancer research and control, European countries should aim for the following:

- Closing “white spots” and achieving full coverage of cancer registration for the whole of Europe.
- Developing precise, standardized recommendations for recording and reporting NMSC that are feasible for implementation across all population-based registries.
- Recording NMSC, at least cSCC, in as many countries as possible, or at least setting up well-defined sentinel regions capturing all NMSCs.
- Better integration of outpatient and pathology data and application of digital and AI-based technologies to improve cancer case detection, registration, and data quality.
- Acceleration of data flows from cancer registries towards the ECIS in the direction of near-real-time reporting in the ECIS.
- Sustainable European collaboration and funding mechanisms to maintain and expand registry infrastructures.

Ultimately, complete, interoperable, and timely registry data will enable Europe to monitor the effectiveness of prevention, guide health policy, and reduce inequalities, not only in skin cancer.

## Figures and Tables

**Table 1:** Estimated absolute numbers and age-standardized incidence rates (ASR, EU-2013) for all cancer sites (excluding non-melanoma skin cancer (NMSC)) and for cutaneous melanoma in the EU Member States in the year 2024. For data sources and methods, see the ECIS [15] (accessed on 6 January 2026).

Country	All Cancer Sites (Excluding NMSC)		Cutaneous Melanoma			
	Both Sexes		Both Sexes		Women	Men
	Number	ASR/ 100,000	Number (% of All Cancers)	ASR/ 100,000	ASR/100,000	ASR/100,000
Austria	46,707	488.0	2213 (4.7%)	23.2	18.4	30.1
Belgium	71,647	595.7	4300 (6.0%)	36.5	39.4	34.3
Bulgaria	30,698	404.7	463 (1.5%)	6.2	5.5	7.3
Croatia	26,710	603.7	1070 (4.0%)	24.6	20.3	31.1
Cyprus	4447	395.7	86 (1.9%)	7.5	7.5	7.9
Czechia	60,918	540.8	3003 (4.9%)	26.8	23.8	32
Denmark	41,325	668.0	3055 (7.4%)	50.7	52.6	49.7
Estonia	8135	578.2	268 (3.3%)	19.3	18.7	21.8
Finland	33,937	536.6	2030 (6.0%)	33.1	29.5	39
France	420,333	581.3	16,075 (3.8%)	22.8	22	24.3
Germany	528,314	549.4	26,449 (5.0%)	28.3	26.7	31.1
Greece	62,467	510.4	1267 (2.0%)	11	9.6	12.8
Hungary	60,402	592	1407 (2.3%)	13.9	13.2	15.8
Ireland	26,183	588.7	1471 (5.6%)	32.8	28.5	38.3
Italy	402,668	555.3	12,997 (3.2%)	19.1	17.2	21.7
Latvia	10,854	537.1	224 (2.1%)	11.3	12.2	11.3
Lithuania	16,077	535.5	374 (2.3%)	12.7	12.5	13.8
Luxembourg	2731	472.7	149 (5.5%)	25.7	19.1	33.2
Malta	2772	512.8	85 (3.1)	15.4	13.7	17.8
Netherlands	112,856	602.3	7822 (6.9%)	42.6	41.6	44.4
Poland	196,396	508.3	3319 (1.7%)	8.6	7.6	10.5
Portugal	66,856	565.5	1280 (1.9%)	11	10.5	11.7
Romania	95,649	489.4	1632 (1.7%)	8.4	7.9	9.4
Slovakia	29,674	563.3	908 (3.1%)	17.1	14	22
Slovenia	13,571	582.2	721 (5.3%)	31.3	29.1	35.2
Spain	263,208	502.1	6257 (2.4%)	12.1	11.1	13.4
Sweden	60,277	554.4	4941 (8.2%)	46.1	43.9	49.2
EU-27	2,695,812	546.6	103,863 (3.9%)	21.5	20	23.9

**Table 2:** Overview of registry-based publications on skin cancer published in PubMed-indexed journals using European cancer registry (CR) data from 2015 to October 2025. Categories are not mutually exclusive. Information retrieved from title and abstract on 22 October 2025.

Search Criteria	Hits		Hits
Publications with “skin cancer” and “cancer registries” on PubMed	1176		
including at least one European CR	538		
including the following keyword		including country name (<20 hits)	
incidence or prevalence	508	Netherlands	77
survival	264	Norway	59
trend	158	Germany	50
treatment	226	Italy	49
operation	42	UK	43
chemotherapy	42	Sweden	41
radiotherapy	65	Denmark	29
NMSC	82	Ireland	24
BCC or SCC	98	Finland	22

## Data Availability

The data underlying this review are predominantly derived from publicly accessible sources, including the European Cancer Information System (ECIS), the Global Cancer Observatory (GCO), national cancer registry reports, and the cited scientific literature. Results from the ENCR Working Group on Non-Melanoma Skin Cancer have not yet been formally published. No new datasets were generated or analyzed for this study.

## Abbreviations

The following abbreviations are used in this manuscript:

CM	Cutaneous Melanoma
NMSC	Non-Melanoma Skin Cancer
BCC	Basal Cell Carcinoma
cSCC	Cutaneous Squamous Cell Carcinoma
ECIS	European Cancer Information System
ENCR	European Network of Cancer Registries
GCO	Global Cancer Observatory
NORDCAN	Association of the Nordic Cancer Registries
